# Progress on neoadjuvant immunotherapy in resectable non-small cell lung cancer and potential biomarkers

**DOI:** 10.3389/fonc.2022.1099304

**Published:** 2023-01-24

**Authors:** Xinyu Wu, Yi Fung Chau, Hua Bai, Xiaofei Zhuang, Jie Wang, Jianchun Duan

**Affiliations:** ^1^ CAMS Key Laboratory of Translational Research on Lung Cancer, State Key Laboratory of Molecular Oncology, Department of Medical Oncology, National Cancer Center/National Clinical Research Center for Cancer/Cancer Hospital, Chinese Academy of Medical Sciences Peking Union Medical College, Beijing, China; ^2^ Department of Thoracic Surgery, Shanxi Province Cancer Hospital/Shanxi Hospital Affiliated to Cancer Hospital, Chinese Academy of Medical Sciences/Cancer Hospital Affiliated to Shanxi Medical University, Taiyuan, China; ^3^ Department of Medical Oncology, Shanxi Province Cancer Hospital/Shanxi Hospital Affiliated to Cancer Hospital, Chinese Academy of Medical Sciences/Cancer Hospital Affiliated to Shanxi Medical University, Taiyuan, China

**Keywords:** non-small cell lung cancer, neoadjuvant therapy, immune checkpoint inhibitors, PD-L1, circulating tumor DNA, tumor mutation burden

## Abstract

Immune checkpoint inhibitors (ICIs) are highly concerned in the treatment of non-small cell lung cancer (NSCLC), represented by inhibitors of programmed death protein 1 (PD-1) and its ligand (PD-L1), and inhibitors of cytotoxic T lymphocyte-associated antigen-4 (CTLA-4). The introduction of immunotherapy in the treatment of perioperative NSCLC has improved the prognosis to a great extent, as demonstrated by several phase II and III clinical trials. The target population for immunotherapy in early-stage NSCLC is still under discussion, and the biomarkers for neoadjuvant immunotherapy population selection are the next pending problem. The predictive efficacy of many potential makers is still being explored, including PD-L1 expression levels, tumor mutation burden, circulating tumor DNA, components of the tumor microenvironment, and several clinical factors. We summarize key findings on the utility of ICIs in clinical trials of preoperative NSCLC patients and conclude analyses of relevant biomarkers to provide a better understanding of potentially predictive biomarkers in neoadjuvant immunotherapy.

## 1 Introduction

Lung cancer is the leading lethal cause of malignant tumors worldwide. In recent decades, randomized trials worldwide have shown a 24-33% reduction in lung cancer mortality through low-dose CT screening in high-risk populations (1, 2). Notably, over 30% of NSCLC patients at diagnosis are considered resectable, including stage I-II and a selective portion of IIIA and IIIB (SEER database, Cancer statistics). For early-stage NSCLC, the best way to optimize patients’ outcomes is radical resection together with proper maintenance treatment. Especially for patients with stage IIB and stage III tumors, who could consider more than one treatment modality (surgery, radiation therapy, or chemotherapy), a multidisciplinary evaluation is usually recommended by The National Comprehensive Cancer Network (NCCN) clinical guidelines, including thoracic surgeons, physicians, radiation oncologists, and pathology oncologists. For patients who undergo successful surgical resection, a significant proportion may face difficult problems such as postoperative complications, local recurrence, and distant metastases, which reduce the quality of life and shorten survival after surgery. Therefore, for patients with stage IB (with high-risk factors) to stage IIIB (operable evaluated by surgeons) NSCLC, the primary issue is radical R0 resection with routine postoperative adjuvant therapy to reduce the probability of postoperative recurrence and prolong disease-free survival (DFS) and overall survival time (OS). Neoadjuvant therapy has shown its powerful ability to downstage and bring curative surgical opportunities to patients with early-stage and locally advanced NSCLC.

Chemotherapy has been the standard of care (SoC) in the adjuvant and neoadjuvant settings in rsectable NSCLC for a long period. Current data shows that neoadjuvant chemotherapy improved the OS by around 5% in OS and time to recurrence in patients with resectable NSCLC (3). The addition of radiation to neoadjuvant chemotherapy did not seem to further improve the survival benefit (4). Radiation gives help to control locoregional disease, but the PFS extension fails to translate into a long-term survival benefit (5–7). Therefore, New strategies aiming for a superior outcome are under exploration.

**Figure 1:**
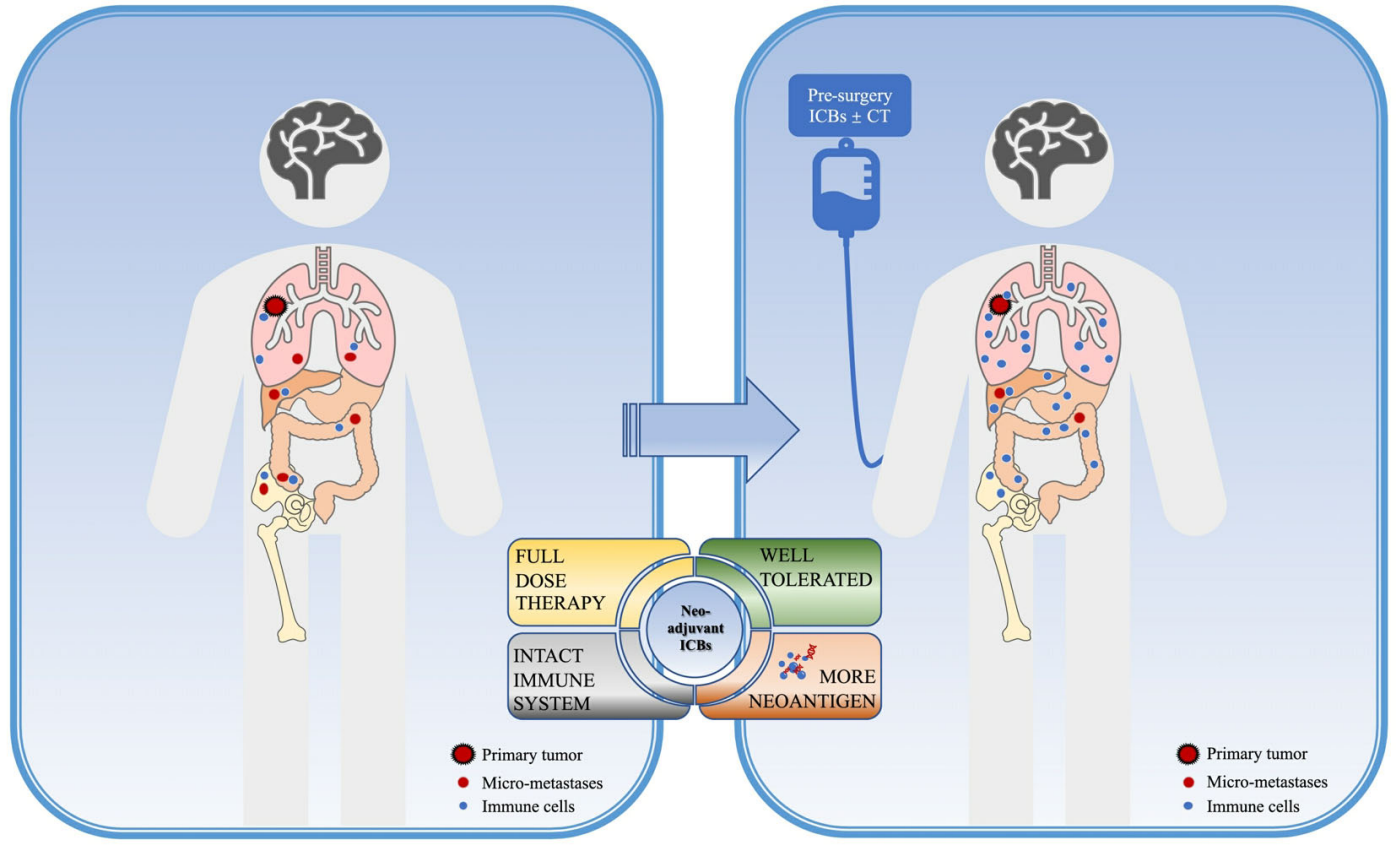
Rationale of neoadjuvant immunotherapy. Neoadjuvant immune checkpoint inhibitors (ICBs) in NSCLC might provide higher benefits for non-small cell lung cancer (NSCLC) patients due to the following points: before surgery, the primary tumor and distant micro-metastases providing a pleural of tumor-specific neoantigens, with the intact immune system, the immune response could be of the greatest work, which could obliterate micro-metastases in return. Also, better performance status before surgery could offer higher opportunities for patients to receive a full-dose systemic therapy with well tolerance. ICB, immune checkpoint inhibitors; CT, chemotherapy.

The rationale for immune neoadjuvant therapy could be concluded as the following points (**Figure 1**): Firstly, the excellent efficacy of immunotherapy in locally advanced and metastatic NSCLC has been confirmed by several clinical trials, and both FDA and NMPA have approved several PD-1/PD-L1/CTLA-4 inhibitors alone or in combination for the first-line treatment of advanced driver-negative NSCLC; secondly, pre-operation patients are more likely to better tolerate full-dose systemic therapy with a better performance status(PS) score and fewer complications. Another reason to support immune neoadjuvant therapy is that preoperative patients harbor a relatively high tumor burden and high neoantigen loads, besides, the immune system remains intact so the application of immunotherapy at this time can maximize the strength and activate the immune system to kill tumor cells and obliterate distant micro-metastases (8). This will provide the basis for tumor shrinkage, down-staging, and more complete radical surgery, to obtain longer survival benefits.

Currently, trials on neoadjuvant immunotherapy for resectable NSCLC patients are on the way. Combinations with chemotherapy and radiation, treatment cycles, and pre-and post-operative distribution, multiple issues are still under discussion. Despite the superior efficacy of neoadjuvant immunotherapy compared to standard chemotherapy, some patients do not benefit from the treatment, progress during treatment, or relapse after surgery. Thus, to monitor the dynamic of cancer disease, select optimized regimens for different populations, and predict response to neoadjuvant therapy, biomarkers involving tumor tissues and peripheral blood are discussed. Here we review the updated data from clinical trials and track the latest exploratory analysis on biomarkers, aiming to provide a better understanding of the routine use of biomarkers in clinical settings.

## 2 Evaluations of neoadjuvant therapy

To better evaluate the effect of tumor treatment, response evaluation criteria in solid tumors (RECIST) was proposed in 2000, and further updated by RECIST version 1.1 (9), which mainly evaluates the change of tumor size before and after treatment by radiological features, thus disease remission, stability or progression was judged. However, due to the limitations of the radiology characteristics, it is not clear whether the changes in images are disease progression or inflammatory response, and the effect of treatment may be underestimated. Along with the development of immunotherapy, immune response evaluation criteria in solid tumors (iRECIST) (10) were proposed to better suit the situation, but all these criteria were not quite fit in the neoadjuvant setting. Given the uniqueness of neoadjuvant therapy, as the primary lesion can be evaluated after surgical resection, pathological features can now be used as one of the approaches to assess the efficacy of neoadjuvant therapy for resectable NSCLC (11). The evaluation of pathological responses consists of a complicated evaluation (11), mainly including assessments of the percentages of (a) viable tumor, (b) necrosis, and (c) stroma (including inflammation and fibrosis) with a total adding up to 100%. It is now widely accepted that pathological response could be a surrogate endpoint of survival in studies of neoadjuvant therapy (12, 13). Previous studies have proved that major pathological response (MPR) defined as no more than 10% viable tumor in resected specimen could be a better predictor of overall survival than overall response rate (14, 15). Pathological complete response (pCR), is uniformly defined as no viable tumor cells after complete evaluation of a resected lung cancer specimen including all sampled regional lymph nodes, which is staged as ypT0N0 in the AJCC system (8^th^ edition). Pathological complete response(pCR) is another good measurement for efficacy, but due to its infrequency in the clinic, MPR is more widely used in both clinical evaluation and as an endpoint in clinical trials.

## 3 Clinical trials on neoadjuvant immunotherapy in potentially resectable NSCLC

### 3.1 Neoadjuvant immune monotherapy

MK3475-223 (16) is a Phase 1 study focused on the safety profile of Pembrolizumab in stage I/II NSCLC, only 6 patients were enrolled in this study, out of which 2 patients shown response. The overall results indicated that pembrolizumab is well tolerated. Another two phase 2 studies, TOP 1501 (17) and NEOMUN (18), further explored the utility of pembrolizumab as a neoadjuvant treatment in different settings of post-operative adjuvant study. Patients in TOP 1501 study received 2 cycles of neoadjuvant pembrolizumab and 4 cycles of adjuvant pembrolizumab with or without radiotherapy or chemotherapy as adjuvant maintenance therapy; while NEOMUN study required standard of care treatment as adjuvant therapy. All three studies suggested that pembrolizumab was safe and well tolerated with a higher pathological response rate compared to neoadjuvant chemotherapy, and not associated with excess surgical morbidity. Checkmate-159 (19) is a prospective phase 2 study, intended to evaluate the patients reached outcome and safety profile using nivolumab for 2 cycles as neoadjuvant monotherapy, followed by resection within 14 days. The outcome was encouraging, with 45% of patients reached MPR, of which 15% reached pCR. Though the population was relatively small (21 patients), it still demonstrated the potential of immunotherapy in pre-operation NSCLC patients. NEOSTAR study (20) enrolled 44 NSCLC patients, and explored mono-nivolumab or combined with ipilimumab followed by surgery. Thirty-nine out of 44 patients underwent surgery, and the R0 resection rate was 100%. No significant difference in pathological and radiological responses was observed between mono- and dual-immunotherapy. ChiCTR-OIC-17013726 (21) is a phase 1b study that evaluated the safety and outcome of sintilimab (a PD-1 inhibitor) in neoadjuvant setting. The study enrolled 40 patients with resectable NSCLC (stage IA–IIIB), including six patients with stage T3N2M0 and two patients with T4N2M0, all of whom received 2 cycles of sintilimab and 37 of whom underwent resection. The MPR rate reached 40.5%, demonstrating a reliable efficacy of neoadjuvant immune-monotherapy. So far, the LCMC3 study (22) recruited the largest population of patients with IB-IIIA(including selected IIIB)resectable NSCLC in the neoadjuvant immunotherapy setting. Patients were given two doses of pre-surgery atezolizumab and a post-operative atezolizumab as maintenance therapy for up to 12 months. A total of 181 patients were enrolled, 159 underwent surgery, out of which 30(20.4%) reached MPR, 10(6.8%) reached pCR. Clinical trials of mono-drug neoadjuvant immunotherapy are summarized in **Table 1**. 

**Table 1:** Clinical trials of neoadjuvant immunotherapy(mono-drug) in NSCLC.

Identifier	Acronym	phase	design	stage	Number of patients	Intervention	1 End Point	Biomarkers
**NCT02938624**	MK3475-223	1	Single arm	I-II	28	Pembrolizumab → Surgery	MPR; Toxicity	NG
**NCT03030131**	IONESCO	2	Single arm	IB-IIIA	46	Durvalumab → Surgery*	R0 resection	NG
**NCT02818920**	TOP 1501	2	Single arm	IB-IIIA	35	Pembrolizumab ×2→ Surgery → Pembrolizumab ×4	Surgical feasibility rate	NG
**NCT03197467**	NEOMUN	2	Single arm	II-IIIA	30	Pembrolizumab → Surgery	Feasibility; Safety; Clinical responses Pathological responses	NG
**NCT02259621**	CheckMate 159	2	Single arm	I-selected IIIB	21	Nivolumab → Surgery	Safety; Feasibility	PD-L1
**NCT02927301**	LCMC3	2	Single arm	IB-IIIB	180	Atezolizumab → Surgery → Atezolizumab	MPR	TMB; WES
**NCT03158129**	NEOSTAR	2	Parallel	I-IIIA	44	Nivolumab, Q2W×3→ Surgery → SoC	MPR	PD-L1; TIL quantification; Blood, tissue, and stool-based biomarkers
						Nivolumab, Q2W×3 + ipilimumab ×1 → Surgery → SoC		
**ChiCTR-OIC-17013726**	/	1b	Single arm	IA–llIB	49	Sintilimab ×2 → Surgery	Safety; Feasibility	PET-CT SUVmax
**NCT02994576**	PRICNEPS	2	Single arm	IA-IIIA (No N2)	60	Atezolizumab → Surgery	Toxicity	NG

* 27 patients received adjuvant therapy (chemotherapy or chemotherapy plus radiotherapy).

NSCLC, Non-small cell lung cancer; SoC, standard of care; NG, not given; MPR, major pathological response; TIL, tumor infiltrating lymphocytes.

### 3.2 Immunotherapy combinations

Immunotherapy combinations have shown better efficacy than monotherapy in neoadjuvant settings (**Table 2**). TOP 1201(NCT01820754) (23) was the first study that demonstrate the safety and feasibility of neoadjuvant immunochemotherapy in resectable NSCLC. In this study, 2 to 3 cycles of ipilimumab combined with chemotherapy were used before surgery. Compared to historical data on neoadjuvant chemotherapy, the postoperative morbidity rate was not worse. NADIM study (24) confined the enrollment to stage IIIAN2 NSCLC patients. It is the earliest study that evaluated the safety, efficacy, and outcome of nivolumab combined with standard chemotherapy in the neoadjuvant setting of NSCLC. The combination of nivolumab and standard chemotherapy shown a high radical surgery rate (41/46, 89%) and a relatively long survival (3-year survival rate: 81.9%), confirming the feasibility of combined therapy in locally advanced NSCLC. Further, NADIM II(NCT03838159) study expanded the population to stage IIIA and IIIB(T3N2) patients, with a total count of 90 patients. Notably, the NADIM II study planned three cycles of nivolumab plus platinum-based doublet chemotherapy before surgery and postoperative maintenance immunotherapy using nivolumab (480mg Q4W) for six months, which is quite different from other adjuvant treatments (adjuvant immunotherapy for 1 year). In 2022 WCLC (25), researchers updated the results of this trial. The surgery outcome favored the immunochemotherapy neoadjuvant strategy, with a R0 resection percentage of 92.5% in the combination group compared to that of chemotherapy group (65%). Also, the downstaging was markable in combination group, nearly 70% (37/53) patients reached a successful downstaging in combination group, where the number is 40% (8/20) in the chemo-group. The intention to treat population for nivo + chemo arm is 56 patients, out of which 21 reached pathological complete remission (pCR, 37.5%). Compared to that of chemo-group (2/28, 7.1%), the pCR rate is significantly higher while also comparable to the previous studies. Subgroup analysis suggested that PD-L1-positive patients and patients reached pCR shall benefit most from the immunochemotherapy neoadjuvant strategy. It is noteworthy that NADIM II study is the first trial that presented OS benefit in resectable stage IIIA-IIIB NSCLC patients. Several studies explored a new PD-1 inhibitor (Toripalimab) combined with chemotherapy as neoadjuvant therapy in stage IIB-IIIC NSCLC patients. In Renaissance Study (26) and ChiCTR1900024014 (27), patients that underwent surgical procedures reached a 100% R0 resection. The MPR and pCR rate was consistent with previous studies, ranging from 40.9% to 62.5% and 18% to 45%, respectively. Another study conducted in China neoSCORE (28) compared the possible difference between two cycles and three cycles of neoadjuvant immunochemotherapy. As the result suggested, there was a numerical but not statistical difference between the two arms, three cycles of neoadjuvant sintilimab plus doublet-chemotherapy shown a better MPR rate numerically. Also, the higher MPR rate benefit was shown in squamous NSCLC than in non-squamous NSCLC(p=0.003), consistent with a former study reported in 2020 by Shu CA. et al. (29), possibly because of a higher tumor necrosis rate in squamous cancer as observed in neoadjuvant chemotherapy cohorts (30).

**Table 2:** Clinical trials of neoadjuvant immunotherapy (combined therapy) in NSCLC.

Identifier	Acronym	phase	design	stage	Number of patients	Intervention	1 End Point	Biomarkers
**NCT01820754**	TOP 1201 IPI	2	Single arm	IB-IIIA	24	CT ×1 + (Ipilimumab + CT) ×2 → Surgery	Percentage of Subjects with Detectable Circulating T Cells After Treatment	NG
**NCT03794544**	NeoCOAST	2	Single arm	I(>2cm) -IIIA	27	Durva ×1 → Surgery	MPR rate	PD-L1; tumor and microbiome biomarkers; blood mRNA signatures
**NCT05061550**	NeoCOAST-2	2	Parallel	II-IIIA	140	Durvalumab + CT ×4+ Oleclumab → Surgery → Durvalumab + Oleclumab	pCR; Safety	PD-L1; ctDNA dynamics; immunogenicity
						Durvalumab + CT + Monalizumab → Surgery → Durvalumab + Monalizumab		
**NCT03081689**	NADIM	2	Single arm	IIIA (N2)	46	Nivolumab + CT, Q3W ×3 → Surgery → Nivolumab (240mg, Q2W×4m; 480mg, Q4W×8m)	24-month PFS	PD-L1; TMB; peripheral blood immune status; ctDNA
**NCT03838159**	NADIM II	2	Parallel	IIIA/IIIB	90	Nivolumab + CT, Q3W ×3 → Surgery → Nivolumab (480mg, Q4W×6m)	pCR	ctDNA
						CT, Q3W ×3 → Surgery		
**NCT04606303**	Renaissance	2	Single arm	IIB-IIIB	53	Toripalimab + CT, Q3W ×2-4 → Surgery	MPR; pCR	NG
**NCT04144608**	TOGATHER	2	Single arm	IIIA-IIIB	40	Toripalimab + CT, Q3W ×2-4 → Surgery → Toripalimab + CT, Q3W ×2, Toripalimab Q3W ×13	R0 resection rate	IHC; RNA-seq; WES; TCR-seq
**NCT04304248**	NeoTAP01	2	Single arm	IIIA-IIIB (T3-4N2)	33	Toripalimab + CT, Q3W ×3 → Surgery	MPR	PD-L1
**NCT04459611**	neoSCORE	2	Parallel	IB-IIIA	60	Sintilimab + CT ×2 → Surgery → CT ×2 + Sintilimab(up to 1 year)	MPR rate	NG
						Sintilimab + CT ×3 → Surgery → CT ×1 + Sintilimab(up to 1 year)		

NSCLC, Non-small cell lung cancer; CT, chemotherapy; NG, not given; pCR, pathological complete response; MPR, major pathological response; EFS, event free survival; IHC, Immunohistochemistry; WES, Whole Exome Sequencing; TCR-seq, T-cell receptor sequencing; ctDNA, circulating tumor DNA; TMB, tumor mutation burden.

Checkmate 816 is the first and only phase III study of neoadjuvant immunochemotherapy in early-stage NSCLC presenting the primary results so far. The trial included 358 patients with resectable NSCLC newly diagnosed as IB-IIIA stage with no known sensitive mutations of EGFR or ALK. Participants were randomly assigned to receive 3 cycles of nivolumab(360mg) plus platinum-based doublet chemotherapy once every three weeks, or chemotherapy alone. In the first analysis, the pCR benefits of adding nivolumab to chemotherapy were attained regardless of the patient’s age or gender, disease stage, histology, PD-L1 expression, and tumor mutation burden. In the further analysis of the other primary endpoint (31), event-free survival (EFS, defined as the length of time from randomization to any disease progression precluding surgery, disease progression or recurrence after surgery, or death due to any cause), the superior efficacy of combined therapy was proved again. The median EFS of combined therapy reached 31.6 months (95%CI, 30.2-NR), which reflected a 10.8 months longer event-free survival as compared to the chemotherapy arm (20.8, 95%CI, 14.0-26.7; HR 0.63, 97.38%CI 0.43-0.91). What’s more, the benefit of pCR was seen in all patients without regard to PD-L1 expression levels, and a significantly prolonged EFS was noticed in the PD-L1≥1% subgroup (HR 0.41, 95%CI 0.24-0.70), especially in PD-L1 ≥50% subgroup (HR 0.24, 95%CI 0.10-0.61). Significant improvement in EFS and pCR supports NIVO+ chemotherapy as a potential new treatment option for patients with resectable non-small cell lung cancer. According to the excellent results of Checkmate-816, nivolumab plus doublet chemotherapy has now been approved by FDA as a neoadjuvant treatment choice for resectable NSCLC in March 2022. This is so far the first neoadjuvant immunochemotherapy regimen approved by FDA.

In AACR 2022, Cascone, T., et al. reported results from the phase 2, randomized multidrug platform study of neoadjuvant durvalumab alone or combined with novel agents in patients with resectable NSCLC(NeoCOAST) (32). Patients with stage I-IIIA NSCLC were given durvalumab alone or combined with the anti-CD73 mAb oleclumab, the anti-NKG2A mAb monalizumab, or the anti-STAT3 antisense oligonucleotide danvatirsen as neoadjuvant therapy for one cycle followed by surgery. The combination has shown improvement in both MPR and pCR rates compared to durvalumab monotherapy with no new safety signals. Another study, NeoCOAST-2 (33), is an open-label, randomized parallel phase 2 study comparing four doses of neoadjuvant durvalumab combined with CT and either oleclumab or monalizumab, followed by surgery and twelve doses of adjuvant durvalumab plus oleclumab or monalizumab, in patients with resectable, Stage IIA-IIIA NSCLC. These data warrant further investigation in resectable NSCLC.

Apart from the Checkmate-816 study mentioned above, there are more phase 3 studies ongoing currently (**Table 3**). The AEGEAN study (34) aimed to evaluate the efficacy and tolerability of Durvalumab plus standard chemotherapy for up to 4 cycles as preoperative treatment in resectable Stage IIA to select (N2) IIIB NSCLC. Study enrollment began in December 2018, with primary completion anticipated in April 2024. KEYNOTE-671 (NCT03425643) (35) is an international randomized, double-blind, placebo-controlled phase 3 study that evaluates standard neoadjuvant chemotherapy with perioperative pembrolizumab or placebo in early-stage NSCLC. An estimated 786 patients will be enrolled. IMpower030(NCT03456063) (36) is a Phase 3, double-blind, randomized study, 374 resectable stage II - select IIIB (T3N2) NSCLC patients will be enrolled, randomized to either 4 cycles of neoadjuvant atezolizumab (1200 mg Q3W, Arm A) or placebo (Arm B) in combination with an platinum-based chemotherapy regimen. Patients in Arm A will receive adjuvant atezolizumab treatment for up to 16 cycles, and patients in Arm B will receive best supportive care. RATIONALE-315 (37) is a dual-primary endpoint phase 3 study, evaluating the efficacy of neoadjuvant tislelizumab or placebo + platinum-based doublet chemotherapy for 3-4 cycles followed by adjuvant tislelizumab or placebo for up to 8 cycles. Results from these trials and more ongoing trials are anticipated for a better understanding of the efficacy and safety of immunotherapy in the neoadjuvant setting of NSCLC. Also, long-term follow-up data could provide more information on the selection of immune checkpoint inhibitors and the most beneficial population.

**Table 3:** Clinical trials of neoadjuvant immunotherapy (phase III randomized clinical trials) in NSCLC.

Identifier	Acronym	stage	Number of patients	Driver gene	Intervention	1 end point	biomarkers
**NCT02998528**	CheckMate-816	IB-IIIA	358	EGFR/ALK WT	Nivolumab+ CT, Q3W → Surgery*	pCR(24%); EFS(31.6m)	ctDNA clearance
					CT, Q3W → Surgery*	pCR(2.2%); EFS(20.8m)	
**NCT03425643**	KEYNOTE-671	II-IIIB (T3-4N2)	786	EGFR/ALK WT	Pembrolizumab + CT → Surgery → Pembrolizumab	EFS; OS	
					CT → Surgery → Placebo		
**NCT03456063**	IMpower-030	II-IIIB (T3N2)	374	EGFR/ALK WT	Atezolizumab + CT, Q3W×4→ Surgery → Atezolizumab Q3W for up to 16 cycles	MPR → EFS	NG
					CT, Q3W×4 → Surgery → Supportive care		
**NCT03800134**	AEGEAN	IIA-IIIA (T3N2)	816	EGFR/ALK WT	Durvalumab + CT, Q3W×3-4 → Surgery → Durvalumab, Q4W×12	pCR; EFS	NG
					CT Q3W×3-4 → Surgery → Placebo		
**NCT04379635**	RATIONALE-315	II-IIIA	380	EGFR/ALK WT	Tislelizumab + CT, Q3W×3-4→ Surgery →Tislelizumab, Q6W×8	MPR; EFS	NG
					CT, Q3W×3-4 → Surgery → Placebo		
**NCT04025879**	CheckMate 77T	II-IIIB	452	EGFR/ALK WT	Nivolumab + CT → Surgery → Nivolumab	EFS	NG
					CT → Surgery → Placebo		
**NCT04158440**	/	II-IIIB (N2)	406	EGFR/ALK WT	Toripalimab + CT, Q3W ×4 → Surgery → Toripalimab + CT, Q3W ×13	MPR; EFS	PD-L1; TMB; WES; ctDNA dynamics
					CT, Q3W ×4 → Surgery → Placebo Q3W ×13		

*: followed by optional adjuvant chemotherapy with or without radiotherapy,

NSCLC, Non-small cell lung cancer; CT, chemotherapy; SoC, standard of care; NG, not given; pCR, pathological complete response; MPR, major pathological response; EFS, event free survival; EGFR, epidermal growth factor receptor; ALK, anaplastic lymphoma kinase; WES, Whole Exome Sequencing; ctDNA, circulating tumor DNA; TMB, tumor mutation burden.

## 4 Potential predictive factors of neoadjuvant immunotherapy

### 4.1 PD-L1

PD-L1 is a co-regulatory molecule expressed on tumor cells that inhibits T-cell-mediated cell death. T cells express the negative regulator PD-1, which binds to ligands including PD-L1 (CD274) or PD-L2 (CD273). In the presence of PD-L1, T-cell activity is suppressed. The antibody inhibited the interaction between PD-1 and PD-L1, thus improving the anti-tumor activity of endogenous T cells. PD-L1 expression is an FDA-approved biomarker for predicting the efficacy of immunotherapy in advanced NSCLC. Several trials have found that high expression of PD-L1 indicates a longer existence in advanced NSCLC (38, 39). Whether PD-L1 status could be a predictive factor of neoadjuvant immunotherapy in NSCLC is still under discussion. Studies reported different results on this issue. In the study NEOSTAR (20), researchers came up with a conclusion that higher pretreatment PD-L1 level is associated with both radiological and pathologic antitumor activity. Higher pre-treatment tumor cell PD-L1 expression evaluated by immunohistochemistry (IHC) was associated with greater pathological responses and fewer residual tumor cells after treatment. However, it is of note that the pathological responses were also observed in PD-L1 negative patients, and the association was not found between post-therapy tumor PD-L1 expression and responses; thus this is still of doubt whether it could be a proper predictor. Checkmate-159 (15) indicated that major pathological response rate was not related to the PD-L1 expression level at diagnosis. Similar results were observed by Shu, CA. et al. (29), as PD-L1 expression did not appear to be predictive of a treatment benefit in neoadjuvant immunochemotherapy.

### 4.2 Tumor mutation burden

Tumor mutation burden refers to the number of somatic mutations per megabase of interrogated genomic sequence in tumor cells, which varies among malignancies. In metastatic NSCLC, the value of TMB as a predictive molecular marker is controversial (40). Theoretically, higher tumor mutation burden is an implication of higher neoantigens which could activate greater anti-tumor immune response (41), thus trigger a better response to immunotherapy. Some studies observed that patients with a high burden of tumor mutations (TMB-High) might respond better to immunotherapy treatments (42, 43), while in other scenarios the predictive value is doubted. Likewise, it is also under discussion whether TMB could be a predictor in the neoadjuvant setting for immunotherapy. In Checkmate-159 (15), of 20 patients who underwent surgical resection, 12 had provided pre-operative tissue for WES sequencing, and 11 underwent complete resections which are sufficient for evaluation. A higher mutation burden detected by whole-exome sequencing was found to be associated with MPR, and the residual tumor rate was found to be inversely related to the sequence alterations. However, this relation was not observed in other studies with more patients (LCMC3, Checkmate 816) (44).

### 4.3 Circulating tumor DNA

Circulating tumor DNA (ctDNA) proved to be a useful predictive biomarker of recurrence and outcome in the advanced NSCLC (45). In trials, researchers found that early clearance of ctDNA was predictive of a better response to treatment and a longer survival time in metastatic non-small cell lung cancer. As recommended by ESMO (46), different clinical scenarios may require different testing strategies. For example, driver-gene testing is required for disease diagnosis, minimal residual disease (MRD) testing after radical resection requires screening for patient-specific alterations, monitoring of patient-specific alterations also helps to identify early recurrence, and more extensive genetic analysis and genome-wide analysis is required at the stage of disease progression to identify mechanisms of drug resistance and select appropriate targeted agents.

In the neoadjuvant setting, the power of ctDNA as a biomarker of immunotherapy is explored in many studies. Current supporting proof comes from Checkmate-816, a phase 3 prospective study that assesses the efficacy of the nivo + chemo regimen in stage IIA-IIIB patients. As it is reported on 2021 ASCO data from the CheckMate-816 trial, investigators collected a portion of blood from the Nivolumab combination chemotherapy group and the chemotherapy group for 3 courses of ctDNA testing. The results shown that the ctDNA clearance rates were 56% and 34% in the Nivolumab combination and chemotherapy groups, respectively. The investigators further conducted a post-screening study on whether ctDNA was cleared and found that in the ctDNA clearance group, the pCR rates were 46% and 13% in the nivolumab combination and chemotherapy groups, respectively, which were significantly higher than the pCR rates of 24% and 2.2% in the unscreened group. The results of this study again suggest that ctDNA clearance rates are highly correlated with pCR and can be used for efficacy prediction of neoadjuvant immunotherapy efficacy. In the NADIM trial (47), lower pretreatment ctDNA levels were associated with improved PFS and OS, while undetectable ctDNA after neoadjuvant therapy was associated with better PFS and OS. Similar results were also found in NADIM II study (48). Another proof was reported by the LCMC3 study (49), indicating that ctDNA could be a predictor of better pathologic response and longer survival. After immunotherapy, greater ctDNA reduction was seen in patients with MPR than those with non-MPR (median log2 fold change −4.8 vs 0.3, P<0.001). Also, post-immunotherapy reduced ctDNA levels were associated with pathologic response (P<0.001, r=0.38) and regression in radiographic tumor size (P<0.001, r=0.42). What’s more, patients that are ctDNA negative after surgery represented a higher 2-year DFS rate compared to those that are ctDNA positive (75% and 40%, respectively; HR, 3.6; 95% CI: 1.0, 13.1; P=0.054). The inclusion of ctDNA assessment in clinical trials may help identify patients who may be cured with surgery and short-term perioperative treatment, thus avoiding expensive and potentially toxic adjuvant therapy.

### 4.4 Tumor environment components

Multiple components in the tumor microenvironment (TME) surround the tumor cells (50). Differential of the components of the TME might give rise to the proliferation of tumor cells or suppress the growth and metastases of the primary tumor. Cytotoxic immune cells recognize tumor cell antigen and kill the tumor cells; macrophages also give hand to this process. However, tumor cells could manipulate suppressive immune cells in the microenvironment to escape from immune surveillance and even transfer it to a tumor-genic environment. With neoadjuvant therapy, doctors are able to analyze the surgery specimen, where changes in TME could be spotted, and some of the dynamics might be associated with tumor regression and patients’ survival.

Three subgroups from the NADIM study were explored for the potential relation between Peripheral Blood Mononuclear Cells (PBMCs) phenotype and the effect of neo-adjuvant chemo-immunotherapy treatment, especially with the degree of pathological response (51). 41 patients were enrolled in this analysis. The activation of CD4 T cells and NK cells and the expression of PD-1 receptor on immune cells were downregulated. A higher decrease in Platelet/Lymphocyte Ratio (PLR) post-neo-adjuvant treatment, a decrease of PD-1 expression in CD4, CD8, and NK cells, as well as a reduction of CD4 T cells and NK cells activation after neoadjuvant treatment, are associated with pCR. In LCMC3 (52), lower frequencies of ILT2^+^ NK cells and ILT2^+^ NK-like T cells in pretreatment peripheral blood were significantly associated with MPR. Immune profiling by flow cytometry has revealed changes after dual-agent ICI treatment in NEOSTAR study (20). Compared to nivolumab mono-agent arm, frequencies of tumor-infiltrating lymphocytes (TILs), tissue-resident memory (T_RM_) T cells, CD103^+^ effector T_RM_ cells, and CD27^-^CD28^+^ effector memory T cells in resected tumors were higher in dual-agent arm, indicating an enhanced T-cell infiltration. But the changes in TILs were not associated with the extent of pathological response.

T cell receptor (TCRs) clonality has been reported to be associated with acquired resistance to ICIs (53); greater TCR intratumor heterogeneity is associated with an elevated risk of recurrence after surgical procedure (54). In the advanced/metastatic NSCLC patients, studies have found that increased PD-1+ CD8+ TCR clonality after ICI treatment had longer PFS (7.3 months vs. 2.6 months, HR, 0.26; 95% CI, 0.08-0.86; P = 0.002) than those with decreased clonality (55). In the NEOSTAR study, peripheral and tumor TCR clonality was not associated with pathological tumor responses (20), though only one case of MPR was viable for analysis. In Checkmate-159, researchers examined the influence of treatment on T cell clone repertoire in the tumor and peripheral blood at the time of resection (56). Decreased residual tumor rate as well as MPR was associated with higher intratumoral TCR clonality. Further analysis suggests that peripheral T cells might serve as an originating compartment of effective antitumor immunity, and the exchange of T-cell clones between tumor and periphery might play a key role in pathological regression. Another cohort which enrolled 236 patients, suggested that higher TCR repertoire homology between the tumor and uninvolved tumor-adjacent lung indicated an inferior survival due to less tumor-specific T cell effect (57).

### 4.5 Clinical factors

The pivotal approach to measure tumor responses is radiology assessment in non-invasive evaluation methods. As aforementioned, criteria including RECIST, RECIST version1.1, and iRECIST are widely used as a uniform assessment. For most clinical trials ongoing, RECIST was used to assess imaging responses. In the NEOSTAR study (20), within patients who achieved MPR, the ratio of partial response (PR) plus complete response (CR) assessed by imaging according to RECIST was 60% in the dual-agent group. In NeoTAP01, RECIST radiological regression was not associated with pathological response. Reduction in SUVmax from baseline to post-neoadjuvant in ^18^F-FDG PET-CT (58) has a significant relation with pathological tumor response. Due to the special effect of immunotherapy, the radiological response is not identical to that in chemotherapy (59). Studies have revealed that regression bed composed by immune-mediated tumor clearance presenting on radiology imaging after neoadjuvant immunotherapy could accounts for the discrepancy of tumor cells between CT-scan image and pathological assessment (59), thus the RECIST criteria are not always accurate in the evaluation of tumor responses, particularly in neoadjuvant immunotherapy. Currently, it is widely accepted that pathological response is more associated with survival than the radiological response in the neoadjuvant immunotherapy regimen.

Another interesting factor that might predict the outcome of neoadjuvant immune-combined therapy is immune-related adverse events (irAEs). In 2022 WCLC (60), an updated Renaissance trial reported the interesting relation between irAEs and outcome of neoadjuvant Toripalimab combined with platinum-based doublet chemotherapy. Five patients experienced grade 2-3 adverse event, out of which 3 patients underwent resection reached pCR with an interval of 8 weeks between surgery and the last dose of neoadjuvant immunochemotherapy, and the other two patients were not suitable for surgery or in the interval also reached clinical complete response or partial response. No extra surgery difficulties nor delays were spotted in these patients.

An ongoing trial initiated by researchers from Peking Union Medical College Hospital focused on the safety and potential biomarkers of Durvalumab in combination with albumin-paclitaxel plus cisplatin/carboplatin for stage IB-IIIA non-small cell lung cancer (NCT04646837). Whole exome sequencing (WES) and NanoString platform-based GEP (gene expression profiling) were implemented to find potential biomarkers. Another to investigate the impact of neoadjuvant immunotherapy on the tumor microenvironment at multiple levels, including genome, transcriptome, PD-1/PD-L1 protein transcription and expression, T cell TCR immunome library, and T cell subpopulation, aiming to provide comprehensive exploratory research evidence on immune mechanisms of neoadjuvant anti-PD-1/L1 therapy in lung cancer.

## 5 Discussion

Neoadjuvant therapy aims at improving the outcome of early-stage and locally advanced NSCLC. The utility of molecular markers in predicting efficacy has not been uniformly agreed upon, thus it is not necessary to select drugs based on molecular testing (61). A recent retrospective study claimed that the dynamics of circulating tumor DNA, defined as relative delta mean variant allele fraction, predicts neoadjuvant immunotherapy efficacy and recurrence-free survival in surgical non-small cell lung cancer patients, as ctDNA dynamics are concordant with pathologic response, demonstrating 100% sensitivity (62). The circulating tumor DNA recurrence preceded radiographic relapse, with a median time of 6.83 months (62). Studies have supported that patient without minimal residue disease (MRD) after surgery have a much lower risk of recurrence, thus suggesting that MRD might be promising to contribute to the refinement of individualized adjuvant therapy and consolidation treatment (63). As it is reported by Zhang, et al. (64), the negative predictive value of longitudinal molecular residual disease is 96.8%, with only 6 patients reoccurred (3.2%). The findings suggested that MRD negative patients might not benefit from the adjuvant study, and longitudinal MRD negative populations are highly possible to be “cured” as indicated by long-term disease-free survival. Another prospective multicenter cohort study, LIBERTI, intending to evaluate the possible association between presence of circulating tumor DNA and the disease-free survival in completely resected phase II-III NSCLC, is now ongoing (65). Further evidence from prospective randomized clinical trials might help better illustrate the utility of ctDNA as a biomarker in NSCLC.

Till now, there has not been a unanimous biomarker for predicting outcomes of neoadjuvant immunotherapy. As a promising predictive biomarker in advanced NSCLC, PD-L1 expression and tumor mutation burden has been considered as highly potential biomarkers in the neoadjuvant setting predicting the outcome of neo-ICI therapy. However, results vary from different trials (15, 20, 29), no solid evidence till now support the predictive efficacy of these two factors. A recent meta-analysis indicated that PD-L1 expression and TMB could be predictive factors for pathological response (66), with higher expression of PD-L1 (≥1% vs <1%) correlated with higher MPR rate and pCR rate (OR = 2.62, P = 0.0006; OR = 2.94, P ≤0.0001, respectively). Previous studies have reported that PD-L1 status defining through three IHC scoring systems (Ventana SP263, Dako 22C3, and Dako 28-8) are highly agreeable with each other (67, 68), and the positive relation between higher PD-L1 expression and better MPR/pCR rate suggesting PD-L1 to be a potential stable predictive factor in neoadjuvant setting for clinical practice. Due to lack of clinical evidence and the technical problems in measuring TMB, it is removed from the recommended panel for metastatic NSCLC in the NCCN guideline (69). The predictive efficacy of TMB is also doubted in neoadjuvant setting with no more evidence than the Checkmate-159 study (15), in which a higher mean mutational burden number was suspected through sequencing in MPR population than in non-MPR population (311 ± 55 vs. 74 ± 60, P = 0.01). The utility of TMB as an ideal sole biomarker remains in doubt until more supportive evidence accumulated.

Novel technologies have given us more approaches to study the environment of tumor environments deeper. Studies revealed other molecular markers that could be predictive of the efficacy of immunotherapy in advanced NSCLC. For example, detected through next-generation sequencing, the apolipoprotein B mRNA-editing enzyme catalytic polypeptide-like, also known as APOBEC, has been reported to predict the efficacy of immunotherapy (70), not only in NSCLC but also in pan-cancer analysis (71). Previous study (72) found that APOBEC signature in metastatic NSCLC is strongly associated with better immune responses, in terms of ORR and PFS. Tumor bulk RNA sequencing in NADIM trial recently revealed that certain tumor environmental gene expression could predict pCR with the AUC >0.9 (73). With innovational technique implying in this area, novel markers including mutational signature (74, 75), intestinal microbiota (76), radiomics (77, 78) are now under analysis in neoadjuvant setting of NSCLC, with the hope to provide us with a deeper understanding of the tumor environment and evolution. These new markers might perform well when combined with existing markers (TMB, PD-L1, Tumor neoantigen burden, etc.) or even reveal a better performance in predicting efficacy of ICIs in near future.

Pathologists’ interpretation directly affects the interpretation accuracy of pathological response evaluation (79, 80). Consistent regulation of pathological interpretation of pathological response is essential in practice, and evaluations must be performed by experienced pathologists with adequate knowledge of pathological characteristics in post-immunotherapy specimens. As the pathological response to immune checkpoint inhibitors alters from that to chemotherapy, standardization of pathologic evaluation and reports on post-neoadjuvant specimens will give rise to an agreement amongst the pathologists, which will be key in accurately predicting outcomes for individual patients and facilitating comparisons in clinical practice (59). Another question on whether MPR rate or pCR rate could be translated into survival benefits is still under discussion, long-term follow-up of clinical trials and prospective real-world studies might give us more evidence on this issue. Currently, different designs of trials added difficulties to the direct comparison of results. So far, there is no agreement on adjuvant therapy after neoadjuvant immunotherapy, thus the designs vary among studies. Another factor that should be considered is the possible use of radiotherapy in the locally advanced NSCLC. The establishment of radiotherapy in the adjuvant setting of locally advanced NSCLC is still under discussion, but there is no doubt that radiation therapy should be discussed by a multidisciplinary team for the proper treatment of locally advanced patients.

## 6 Conclusion

Up to this date, no molecular marker has been unanimously agreed on as a powerful predictor in neoadjuvant setting. We are expecting a wide range of immunotherapy and combined regimens as well as a more profound genre of predictive and prognostic biomarkers in neoadjuvant setting of NSCLC in the coming future. Dynamic change of circulating tumor DNA is currently the most likely predictive biomarker of neo-immunotherapy. The predicting power of PD-L1 expression warrants further validation, while TMB is not recommended yet. Further exploration on biomarkers focusing on immune-related adverse events is of great importance as well for the underlying population that might not benefit from neoadjuvant immunotherapy.

## Author contributions

XW: Data curation, Writing- Original draft preparation, Visualization. YC: Data curation, Validation. HB: Validation, Supervision. XZ: Reviewing the Final draft. JW: Supervision, Reviewing the Final draft. JD: Conceptualization, Supervision, Reviewing and Editing the Original and Final draft. All authors contributed to the article and approved the submitted version.

## References

[1] de Koning HJ van der Aalst CM de Jong PA Scholten ET Nackaerts K Heuvelmans MA. Reduced lung-cancer mortality with volume CT screening in a randomized trial. N Engl J Med (2020) 382(6):503–13. doi: 10.1056/NEJMoa1911793 31995683

[2] Li N Tan F Chen W Dai M Wang F Shen S. One-off low-dose CT for lung cancer screening in China: A multicentre, population-based, prospective cohort study. Lancet Respir Med (2022) 10(4):378–91. doi: 10.1016/S2213-2600(21)00560-9 35276087

[3] NSCLC Meta-analysis Collaborative Group Burdett S Rydzewska LHM Tierney JF Auperin A Le Pechoux C. Preoperative chemotherapy for non-small-cell lung cancer: a systematic review and meta-analysis of individual participant data. Lancet (2014) 383(9928):1561–71. 10.1016/S0140-6736(13)62159-5 PMC4022989 24576776

[4] Levy A Mercier O Le Péchoux C. Indications and parameters around postoperative radiation therapy for lung cancer. J Clin Oncol (2022) 40(6):556–66. doi: 10.1200/JCO.21.01774 34985927

[5] Uy KL Darling G Xu W Yi QL De Perrot M Pierre AF. Improved results of induction chemoradiation before surgical intervention for selected patients with stage IIIA-N2 non-small cell lung cancer. J Thorac Cardiovasc Surg (2007) 134(1):188–93. doi: 10.1016/j.jtcvs.2007.01.078 17599507

[6] Le Pechoux C Pourel N Barlesi F Lerouge D Antoni D Lamezec B. Postoperative radiotherapy versus no postoperative radiotherapy in patients with completely resected non-small-cell lung cancer and proven mediastinal N2 involvement (Lung ART): an open-label, randomised, phase 3 trial. Lancet Oncol (2022) 23(1):104–14. doi: 10.1016/S1470-2045(21)00606-9 34919827

[7] Hui Z Men Y Hu C Kang J Sun X Bi N. Effect of postoperative radiotherapy for patients with pIIIA-N2 non-small cell lung cancer after complete resection and adjuvant chemotherapy: The phase 3 PORT-c randomized clinical trial. JAMA Oncol (2021) 7(8):1178–85. doi: 10.1001/jamaoncol.2021.1910 PMC8227450 34165501

[8] Janjigian YY Wolchok JD Ariyan CE. Eradicating micrometastases with immune checkpoint blockade: Strike while the iron is hot. Cancer Cell (2021) 39(6):738–42. doi: 10.1016/j.ccell.2021.05.013 34129818

[9] Schwartz LH Litière S de Vries E Ford R Gwyther S Mandrekar S. RECIST 1.1-update and clarification: From the RECIST committee. Eur J Cancer (2016) 62:132–7. doi: 10.1016/j.ejca.2016.03.081 PMC5737828 27189322

[10] Seymour L Bogaerts J Perrone A Ford R Schwartz LH Mandrekar S. iRECIST: Guidelines for response criteria for use in trials testing immunotherapeutics. Lancet Oncol (2017) 18(3):e143–e52. doi: 10.1016/S1470-2045(17)30074-8 PMC5648544 28271869

[11] Travis WD Dacic S Wistuba I Sholl L Adusumilli P Bubendorf L. IASLC multidisciplinary recommendations for pathologic assessment of lung cancer resection specimens after neoadjuvant therapy. J Thorac Oncol (2020) 15(5):709–40. doi: 10.1016/j.jtho.2020.01.005 PMC8173999 32004713

[12] Pataer A Kalhor N Correa AM Raso MG Erasmus JJ Kim ES. Histopathologic response criteria predict survival of patients with resected lung cancer after neoadjuvant chemotherapy. J Thorac Oncol (2012) 7(5):825–32. doi: 10.1097/JTO.0b013e318247504a PMC3465940 22481232

[13] Hellmann MD Chaft JE William WN Jr. Rusch V Pisters KM Kalhor N. Pathological response after neoadjuvant chemotherapy in resectable non-small-cell lung cancers: Proposal for the use of major pathological response as a surrogate endpoint. Lancet Oncol (2014) 15(1):e42–50. doi: 10.1016/S1470-2045(13)70334-6 PMC4734624 24384493

[14] Chen X Ma K. Neoadjuvant therapy in lung cancer: What is most important: Objective response rate or major pathological response? Curr Oncol (2021) 28(5):4129–38. doi: 10.3390/curroncol28050350 PMC8535119 34677268

[15] Forde PM Chaft JE Smith KN Anagnostou V Cottrell TR Hellmann MD. Neoadjuvant PD-1 blockade in resectable lung cancer. N Engl J Med (2018) 378(21):1976–86. doi: 10.1056/NEJMoa1716078 PMC6223617 29658848

[16] Ben Nun A Golan N Ofek E Urban D Kamer I Simansky D. Neoadjuvant pembrolizumab (Pembro) for early stage non-small cell lung cancer (NSCLC): Initial report of a phase I study, MK3475-223. Ann Oncol (2018) 29:viii486. doi: 10.1093/annonc/mdy290.011

[17] Tong BC Gu L Wang X Wigle DA Phillips JD Harpole DH Jr.. Perioperative outcomes of pulmonary resection after neoadjuvant pembrolizumab in patients with non-small cell lung cancer. J Thorac Cardiovasc Surg (2022) 163(2):427–36. doi: 10.1016/j.jtcvs.2021.02.099 33985811

[18] Eichhorn F Klotz LV Bischoff H Thomas M Lasitschka F Winter H. Neoadjuvant anti-programmed death-1 immunotherapy by pembrolizumab in resectable nodal positive stage II/IIIa non-small-cell lung cancer (NSCLC): the NEOMUN trial. BMC Cancer. (2019) 19(1):413. doi: 10.1186/s12885-019-5624-2 31046714 PMC6498462

[19] Reuss JE Smith KN Anagnostou V Zhang J Zahurak M Caushi J. Neoadjuvant nivolumab in resectable non-small cell lung cancer: Extended follow-up and molecular markers of response. J Clin Oncol (2019) 37(15_suppl):8524. doi: 10.1200/JCO.2019.37.15_suppl.8524

[20] Cascone T William WN Jr. Weissferdt A Leung CH Lin HY Pataer A. Neoadjuvant nivolumab or nivolumab plus ipilimumab in operable non-small cell lung cancer: the phase 2 randomized NEOSTAR trial. Nat Med (2021) 27(3):504–14. doi: 10.1038/s41591-020-01224-2 PMC8818318 33603241

[21] Gao S Li N Gao S Xue Q Ying J Wang S. Neoadjuvant PD-1 inhibitor (Sintilimab) in NSCLC. J Thorac Oncol (2020) 15(5):816–26. doi: 10.1016/j.jtho.2020.01.017 32036071

[22] Lee J Chaft J Nicholas A Patterson A Waqar S Toloza E. PS01.05 surgical and clinical outcomes with neoadjuvant atezolizumab in resectable stage IB–IIIB NSCLC: LCMC3 trial primary analysis. J Thorac Oncol (2021) 16(3):S59–61. doi: 10.1016/j.jtho.2021.01.320

[23] Yang CJ McSherry F Mayne NR Wang X Berry MF Tong B. Surgical outcomes after neoadjuvant chemotherapy and ipilimumab for non-small cell lung cancer. Ann Thorac Surg (2018) 105(3):924–9. doi: 10.1016/j.athoracsur.2017.09.030 29258674

[24] Provencio M Nadal E Insa A García-Campelo MR Casal-Rubio J Dómine M. Neoadjuvant chemotherapy and nivolumab in resectable non-small-cell lung cancer (NADIM): an open-label, multicentre, single-arm, phase 2 trial. Lancet Oncol (2020) 21(11):1413–22. doi: 10.1016/S1470-2045(20)30453-8 32979984

[25] Provencio M Serna R Nadal E Glez Larriba JL Martínez-Martí A Bernabé R. PL03.12 progression free survival and overall survival in NADIM II study. J Thorac Oncol (2022) 17(9):S2–3. doi: 10.1016/j.jtho.2022.07.014

[26] Yan S Chen J Wang J Lv C Bi J Yang X. 64P neoadjuvant toripalimab plus chemotherapy in patients with potentially resectable non-small cell lung cancer: A prospective, single-arm, phase II trial (Renaissance study). Ann Oncol (2021) 32:S1400. doi: 10.1016/j.annonc.2021.10.082

[27] Zhu X Sun L Song N Sun F Yang J Duan L. 1176P neoadjuvant PD-1 inhibitor (toripalimab) plus chemotherapy in patients with potentially resectable NSCLC: An open-label, single-arm, phase II trial. Ann Oncol (2021) 32:S942. doi: 10.1016/j.annonc.2021.08.1780

[28] Qiu F Fan J Shao M Yao J Zhao L Zhu L. Two cycles versus three cycles of neoadjuvant sintilimab plus platinum-doublet chemotherapy in patients with resectable non-small-cell lung cancer (neoSCORE): A randomized, single center, two-arm phase II trial. J Clin Oncol (2022) 40(16_suppl):8500. doi: 10.1200/JCO.2022.40.16_suppl.8500

[29] Shu CA Gainor JF Awad MM Chiuzan C Grigg CM Pabani A. Neoadjuvant atezolizumab and chemotherapy in patients with resectable non-small-cell lung cancer: An open-label, multicentre, single-arm, phase 2 trial. Lancet Oncol (2020) 21(6):786–95. doi: 10.1016/S1470-2045(20)30140-6 32386568

[30] Qu Y Emoto K Eguchi T Aly RG Zheng H Chaft JE. Pathologic assessment after neoadjuvant chemotherapy for NSCLC: Importance and implications of distinguishing adenocarcinoma from squamous cell carcinoma. J Thorac Oncol (2019) 14(3):482–93. doi: 10.1016/j.jtho.2018.11.017 PMC6382593 30503889

[31] Forde PM Spicer J Lu S Provencio M Mitsudomi T Awad MM. Neoadjuvant nivolumab plus chemotherapy in resectable lung cancer. N Engl J Med (2022) 386(21):1973–85. doi: 10.1056/NEJMoa2202170 PMC9844511 35403841

[32] Cascone T García-Campelo R Spicer J Weder W Daniel D Spigel D. Abstract CT011: NeoCOAST: open-label, randomized, phase 2, multidrug platform study of neoadjuvant durvalumab alone or combined with novel agents in patients (pts) with resectable, early-stage non-small-cell lung cancer (NSCLC). Cancer Res (2022) 82(12_Supplement):CT011–CT. doi: 10.1158/1538-7445.AM2022-CT011

[33] Cascone T Spira A Campelo RG Kim DW Hamid O Soo-Hoo Y. NeoCOAST-2: A randomized, open-label, phase 2 study of neoadjuvant durvalumab plus novel immunotherapies and chemotherapy (CT) followed by adjuvant durvalumab plus novel agents, in patients with resectable non-small-cell lung cancer (NSCLC). Cancer Res (2022) 82(12_Supplement):CT124. doi: 10.1158/1538-7445.AM20

[34] Heymach JV Mitsudomi T Harpole D Aperghis M Jones S Mann H. Design and rationale for a phase III, double-blind, placebo-controlled study of neoadjuvant Durvalumab + Chemotherapy followed by adjuvant durvalumab for the treatment of patients with resectable stages II and III non-small-cell lung cancer: The AEGEAN trial. Clin Lung Cancer. (2022) 23(3):e247–e51. doi: 10.1016/j.cllc.2021.09.010 34819266

[35] Tsuboi M Luft A Ursol G Kato T Levchenko E Eigendorff E. Perioperative pembrolizumab + platinum-based chemotherapy for resectable locally advanced non-small cell lung cancer: The phase III KEYNOTE-671 study. Ann Oncol (2020) 31:S801–S2. doi: 10.1016/j.annonc.2020.08.1437

[36] Peters S Kim AW Solomon B Gandara DR Dziadziuszko R Brunelli A. IMpower030: Phase III study evaluating neoadjuvant treatment of resectable stage II-IIIB non-small cell lung cancer (NSCLC) with atezolizumab (atezo) 1 chemotherapy. Ann Oncol (2019) 30:ii30. doi: 10.1093/annonc/mdz064.014

[37] Wang C Wang R Ma Y Liang X. TiP neoadjuvant tislelizumab or placebo + platinum-based chemotherapy followed by adjuvant tislelizumab or placebo in patients with resectable non-small cell lung cancer (NSCLC): A phase III trial in progress. J Thorac Oncol (2021) 16(4):S746. doi: 10.1016/S1556-0864(21)01936-5

[38] Gadgeel S Rodríguez-Abreu D Speranza G Esteban E Felip E Dómine M. Updated analysis from KEYNOTE-189: Pembrolizumab or placebo plus pemetrexed and platinum for previously untreated metastatic nonsquamous non-Small-Cell lung cancer. J Clin Oncol (2020) 38(14):1505–17. doi: 10.1200/JCO.19.03136 32150489

[39] Akinboro O Larkins E Pai-Scherf LH Mathieu LN Ren Y Cheng J. FDA Approval summary: Pembrolizumab, atezolizumab, and cemiplimab-rwlc as single agents for first-line treatment of Advanced/Metastatic PD-L1-High NSCLC. Clin Cancer Res (2022) 28(11):2221–8. doi: 10.1158/1078-0432.CCR-21-3844 35101885

[40] Addeo A Friedlaender A Banna GL Weiss GJ. TMB or not TMB as a biomarker: That is the question. Crit Rev Oncol Hematol (2021) 163:103374. doi: 10.1016/j.critrevonc.2021.103374 34087341

[41] Sesma A Pardo J Cruellas M. From tumor mutational burden to blood T cell receptor: looking for the best predictive biomarker in lung cancer treated with immunotherapy. Cancers (Basel) (2020) 12(10):2974. doi: 10.3390/cancers12102974 33066479 PMC7602200

[42] Marabelle A Fakih M Lopez J Shah M Shapira-Frommer R Nakagawa K. Association of tumour mutational burden with outcomes in patients with advanced solid tumours treated with pembrolizumab: Prospective biomarker analysis of the multicohort, open-label, phase 2 KEYNOTE-158 study. Lancet Oncol (2020) 21(10):1353–65. doi: 10.1016/S1470-2045(20)30445-9 32919526

[43] Strickler JH Hanks BA Khasraw M. Tumor mutational burden as a predictor of immunotherapy response: Is more always better? Clin Cancer Res (2021) 27(5):1236–41. doi: 10.1158/1078-0432.CCR-20-3054 PMC9912042 33199494

[44] Forde PM Spicer J Lu S Provencio M Mitsudomi T Awad MM. Nivolumab (NIVO) + platinum-doublet chemotherapy (chemo) vs chemo as neoadjuvant treatment (tx) for resectable (IB-IIIA) non-small cell lung cancer (NSCLC) in the phase 3 CheckMate 816 trial. Cancer Res (2021) 81(13 SUPPL):CT003. doi: 10.1158/1538-7445.AM2021-CT003

[45] Jee J Lebow ES Yeh R Das JP Namakydoust A Paik PK. Overall survival with circulating tumor DNA-guided therapy in advanced non-small-cell lung cancer. Nat Med (2022) 28(11):2353–63. doi: 10.1038/s41591-022-02047-z PMC10338177 36357680

[46] Heitzer E van den Broek D Denis MG Hofman P Hubank M Mouliere F. Recommendations for a practical implementation of circulating tumor DNA mutation testing in metastatic non-small-cell lung cancer. ESMO Open (2022) 7(2):100399. doi: 10.1016/j.esmoop.2022.100399 35202954 PMC8867049

[47] Provencio M Serna-Blasco R Nadal E Insa A García-Campelo MR Casal Rubio J. Overall survival and biomarker analysis of neoadjuvant nivolumab plus chemotherapy in operable stage IIIA non-Small-Cell lung cancer (NADIM phase II trial). J Clin Oncol (2022) 40(25):2924–33. doi: 10.1200/JCO.21.02660 PMC9426809 35576508

[48] Romero A Serna R Nadal E Larriba JLG Martínez-Martí A Bernabé R. MA06.03 pre-treatment ctDNA levels significantly predicts of OS and PFS in NADIM II trial. J Thorac Oncol (2022) 17(9):S63. doi: 10.1016/j.jtho.2022.07.106

[49] Kris MG Grindheim JM Chaft JE Lee JM Johnson BE Rusch VW. 1O dynamic circulating tumour DNA (ctDNA) response to neoadjuvant (NA) atezolizumab (atezo) and surgery (surg) and association with outcomes in patients (pts) with NSCLC. Ann Oncol (2021) 32:S1373. doi: 10.1016/j.annonc.2021.10.017

[50] Quail DF Joyce JA. Microenvironmental regulation of tumor progression and metastasis. Nat Med (2013) 19(11):1423–37. doi: 10.1038/nm.3394 PMC3954707 24202395

[51] Laza-Briviesca R Cruz-Bermudez A Casarrubios M Nadal E Insa A Campelo RG. P2.04-10 biomarkers of pathological response on neo-adjuvant chemo-immunotherapy treatment for resectable stage IIIA NSCLC patients. J Thorac Oncol (2019) 14(10, Supplement):S711. doi: 10.1016/j.jtho.2019.08.1515

[52] Oezkan F Seweryn M Pietrzak M Byun WY Owen D Schulze K. MA09.01 LCMC3: Immune cell subtypes predict nodal status and pathologic response after neoadjuvant atezolizumab in resectable NSCLC. J Thorac Oncol (2021) 16(10):S910–S1. doi: 10.1016/j.jtho.2021.08.152

[53] Anagnostou V Smith KN Forde PM Niknafs N Bhattacharya R White J. Evolution of neoantigen landscape during immune checkpoint blockade in non-small cell lung cancer. Cancer Discovery (2017) 7(3):264–76. doi: 10.1158/2159-8290.CD-16-0828 PMC5733805 28031159

[54] Reuben A Gittelman R Gao J Zhang J Yusko EC Wu CJ. TCR repertoire intratumor heterogeneity in localized lung adenocarcinomas: An association with predicted neoantigen heterogeneity and postsurgical recurrence. Cancer Discovery (2017) 7(10):1088–97. doi: 10.1158/2159-8290.CD-17-0256 PMC5628137 28733428

[55] Han J Duan J Bai H Wang Y Wan R Wang X. TCR repertoire diversity of peripheral PD-1(+)CD8(+) T cells predicts clinical outcomes after immunotherapy in patients with non-small cell lung cancer. Cancer Immunol Res (2020) 8(1):146–54. doi: 10.1158/2326-6066.CIR-19-0398 31719056

[56] Zhang J Ji Z Caushi JX El Asmar M Anagnostou V Cottrell TR. Compartmental analysis of T-cell clonal dynamics as a function of pathologic response to neoadjuvant PD-1 blockade in resectable non-small cell lung cancer. Clin Cancer Res (2020) 26(6):1327–37. doi: 10.1158/1078-0432.CCR-19-2931 PMC7073288 31754049

[57] Reuben A Zhang J Chiou SH Gittelman RM Li J Lee WC. Comprehensive T cell repertoire characterization of non-small cell lung cancer. Nat Commun (2020) 11(1):603. doi: 10.1038/s41467-019-14273-0 32001676 PMC6992630

[58] Tao X Li N Wu N He J Ying J Gao S. The efficiency of (18)F-FDG PET-CT for predicting the major pathologic response to the neoadjuvant PD-1 blockade in resectable non-small cell lung cancer. Eur J Nucl Med Mol Imaging. (2020) 47(5):1209–19. doi: 10.1007/s00259-020-04711-3 PMC7101299 32043180

[59] Cottrell TR Thompson ED Forde PM Stein JE Duffield AS Anagnostou V. Pathologic features of response to neoadjuvant anti-PD-1 in resected non-small-cell lung carcinoma: a proposal for quantitative immune-related pathologic response criteria (irPRC). Ann Oncol (2018) 29(8):1853–60. doi: 10.1093/annonc/mdy218 PMC6096736 29982279

[60] Yan S Chen J Wang J Lv C Bi J Yang X. EP05.02-014 neoadjuvant toripalimab combination in patients with stage IIB-IIIB NSCLC: A single-arm, phase 2 trial (Renaissance study). J Thorac Oncol (2022) 17(9):S288–S9. doi: 10.1016/j.jtho.2022.07.496

[61] Liang W Cai K Chen C Chen H Chen Q Fu J. Expert consensus on neoadjuvant immunotherapy for non-small cell lung cancer. Trans Lung Cancer Res (2020) 9(6):2696–715. doi: 10.21037/tlcr-2020-63 PMC7815365 33489828

[62] Yue D Liu W Chen C Zhang T Ma Y Cui L. Circulating tumor DNA predicts neoadjuvant immunotherapy efficacy and recurrence-free survival in surgical non-small cell lung cancer patients. Transl Lung Cancer Res (2022) 11(2):263–76. doi: 10.21037/tlcr-22-106 PMC8902085 35280315

[63] Moding EJ Nabet BY Alizadeh AA Diehn M. Detecting liquid remnants of solid tumors: Circulating tumor DNA minimal residual disease. Cancer Discovery (2021) 11(12):2968–86. doi: 10.1158/2159-8290.CD-21-0634 PMC8976700 34785539

[64] Zhang JT Liu SY Gao W Liu SM Yan HH Ji L. Longitudinal undetectable molecular residual disease defines potentially cured population in localized non-small cell lung cancer. Cancer Discovery (2022) 12(7):1690–701. doi: 10.1158/2159-8290.CD-21-1486 PMC9394392 35543554

[65] Morgensztern D Green E King JC Sable-Hunt A Erwin R Moore A. Abstract A52: Role of circulating tumor DNA (ctDNA) from liquid biopsy in early-stage NSCLC resected lung tumor investigation (LIBERTI). Clin Cancer Res (2020) 26(11_Supplement):A52–A. doi: 10.1158/1557-3265.LiqBiop20-A52

[66] Deng H Zhao Y Cai X Chen H Cheng B Zhong R. PD-L1 expression and tumor mutation burden as pathological response biomarkers of neoadjuvant immunotherapy for early-stage non-small cell lung cancer: A systematic review and meta-analysis. Crit Rev In Oncology/hematol (2022) 170:103582. doi: 10.1016/j.critrevonc.2022.103582 35031441

[67] Chan AWH Tong JHM Kwan JSH Chow C Chung LY Chau SL. Assessment of programmed cell death ligand-1 expression by 4 diagnostic assays and its clinicopathological correlation in a large cohort of surgical resected non-small cell lung carcinoma. Modern Pathol (2018) 31(9):1381–90. doi: 10.1038/s41379-018-0053-3 29713040

[68] Ratcliffe MJ Sharpe A Midha A Barker C Scott M Scorer P. Agreement between programmed cell death ligand-1 diagnostic assays across multiple protein expression cutoffs in non-small cell lung cancer. Clin Cancer Res (2017) 23(14):3585–91. doi: 10.1158/1078-0432.CCR-16-2375 28073845

[69] Ettinger DS Wood DE Aisner DL Akerley W Bauman JR Bharat A. NCCN guidelines insights: Non-small cell lung cancer, version 2.2021. J Natl Compr Canc Netw (2021) 19(3):254–66. doi: 10.6004/jnccn.2021.0013 33668021

[70] Wang S Jia M He Z Liu X-S. APOBEC3B and APOBEC mutational signature as potential predictive markers for immunotherapy response in non-small cell lung cancer. Oncogene (2018) 37(29):3924–36. doi: 10.1038/s41388-018-0245-9 PMC6053356 29695832

[71] Guo H Zhu L Huang L Sun Z Zhang H Nong B. APOBEC alteration contributes to tumor growth and immune escape in pan-cancer. Cancers (2022) 14(12):2827. doi: 10.3390/cancers14122827 35740493 PMC9221198

[72] Chen H Chong W Teng C Yao Y Wang X Li X. The immune response-related mutational signatures and driver genes in non-small-cell lung cancer. Cancer Science. (2019) 110(8):2348–56. doi: 10.1111/cas.14113 PMC6676111 31222843

[73] Casarrubios M Provencio M Nadal E Insa A Del Rosario García-Campelo M Lázaro-Quintela M. Tumor microenvironment gene expression profiles associated to complete pathological response and disease progression in resectable NSCLC patients treated with neoadjuvant chemoimmunotherapy. J For Immunother Cancer (2022) 10:e005320. doi: 10.1136/jitc-2022-005320 PMC9528578 36171009

[74] Zhou Z Ding Z Yuan J Shen S Jian H Tan Q. Homologous recombination deficiency (HRD) can predict the therapeutic outcomes of immuno-neoadjuvant therapy in NSCLC patients. J Hematol Oncol (2022) 15(1):62. doi: 10.1186/s13045-022-01283-7 35585646 PMC9118717

[75] Rocha P Zhang J Laza-Briviesca R Cruz-Bermúdez A Bota-Rabassedas N Sanchez-Espiridon B. Distinct immune gene programs associated with host tumor immunity, neoadjuvant chemotherapy, and chemoimmunotherapy in resectable NSCLC. Clin Cancer Res (2022) 28(11):2461–73. doi: 10.1158/1078-0432.CCR-21-3207 PMC9167789 35394499

[76] Derosa L Routy B Thomas AM Iebba V Zalcman G Friard S. Intestinal akkermansia muciniphila predicts clinical response to PD-1 blockade in patients with advanced non-small-cell lung cancer. Nat Med (2022) 28(2):315–24. doi: 10.1038/s41591-021-01655-5 PMC9330544 35115705

[77] Kneer K Yousefzadeh-Nowshahr E Raacke J Rodiger S Kropf-Sanchen C Beer M. Image-based biomarkers for low-risk NSCLC using texture analysis of f-18-FDG-PET/CT: Determination of optimal parameters and their prognostic threshold. J Nucl Med (2019) 60.

[78] Mu W Jiang L Shi Y Tunali I Gray JE Katsoulakis E. Non-invasive measurement of PD-L1 status and prediction of immunotherapy response using deep learning of PET/CT images. J For Immunother Cancer (2021) 9(6):e002118. doi: 10.1136/jitc-2020-002118 PMC8211060 34135101

[79] Li L Ling Y Guo C Guo L Ying J. Necrosis is not the main part of immune-related pathologic response to neoadjuvant immunotherapy in squamous cell lung cancer. J Thorac Oncol (2021) 16(1):e7–9. doi: 10.1016/j.jtho.2020.03.032 33384061

[80] Travis WD Dacic S Sholl LM Wistuba II. Pathologic assessment of lung squamous cell carcinoma after neoadjuvant immunotherapy. J Thorac Oncol (2021) 16(1):e9-e10. doi: 10.1016/j.jtho.2020.11.009 33384062

